# Subclass-Aware Contrastive Semi-Supervised Learning for Inflammatory Bowel Disease Classification from Colonoscopy Images

**DOI:** 10.3390/bioengineering13010008

**Published:** 2025-12-22

**Authors:** Kechen Lin, Guangcong Ruan, Xiaoyang Zou, Yongjian Nian, Yanling Wei, Guoyan Zheng

**Affiliations:** 1 Department of Gastroenterology, Daping Hospital, Army Medical University (Third Military Medical University), Chongqing 400042, China; lkkxixixi@sjtu.edu.cn (K.L.); ruanguangcong@tmmu.edu.cn (G.R.); 2 Institute of Medical Robotics, School of Biomedical Engineering, Shanghai Jiao Tong University, Shanghai 200240, China; xiaoyang.zou@sjtu.edu.cn; 3 Department of Digital Medicine, School of Biomedical Engineering and Imaging Medicine, Army Medical University (Third Military Medical University), Chongqing 400038, China; yongjian_nian@163.com

**Keywords:** inflammatory bowel disease, colonoscopy images, semi-supervised learning, contrastive learning

## Abstract

Inflammatory bowel disease (IBD) includes Crohn’s disease (CD) and ulcerative colitis (UC). The accurate classification of IBD from colonoscopy images is critical for diagnosis and treatment. However, the lack of labeled data poses a major challenge for developing deep learning-based IBD classification approaches. Recently, pseudo-labeling-based semi-supervised learning methods offer a promising solution in leveraging both labeled and unlabeled data to improve classification performance. Nevertheless, due to significant intra-class variability and the subtle inter-class differences in IBD colonoscopy images, pseudo-labels are often inaccurate, which results in confirmation bias and suboptimal performance. To address this challenge, a Subclass-Aware Contrastive Semi-Supervised Learning method, referred to as SACSSL, is proposed for accurate IBD classification by integrating a subclass-aware contrastive module into a pseudo-labeling-based semi-supervised framework, e.g., FixMatch. Specifically, unlabeled samples are first partitioned into confident and uncertain samples according to the confidence of pseudo-labels. An instance-level contrastive loss is then applied to uncertain samples, aiming to mitigate confirmation bias. Furthermore, intra-class heterogeneity is captured by introducing a set of prototypes for each subclass and assigning confident samples to these prototypes to form fine-grained subclasses, and supervised contrastive loss is applied to promote intra-subclass clustering, thereby enhancing inter-class separability while preserving intra-class diversity. Our method is evaluated on two datasets, i.e., an in-house collected Daping dataset for IBD classification and a publicly available LIMUC dataset for UC severity grading. On both datasets, our method achieves state-of-the-art performance under the semi-supervised setting. Specifically, with only 20% labeled data, the proposed method reaches an overall accuracy of 93.2% and an F1-score of 80.1% on the Daping dataset, which is close to the fully supervised upper bound (94.0% accuracy and 80.8% F1-score), and it achieves an overall accuracy of 76.4% and an F1-score of 68.9% on the LIMUC dataset. Comprehensive experimental results demonstrate the effectiveness of our method for semi-supervised colonoscopy image classification.

## 1. Introduction

Inflammatory bowel disease (IBD), including Crohn’s disease (CD) and ulcerative colitis (UC) [1], has emerged as a global health concern in recent years [2]. Recent epidemiological studies have shown that IBD has evolved across different global regions with increasing incidence in newly industrialized countries [3]. The symptoms of IBD includes diarrhea, abdominal pain, and rectal bleeding, which can seriously affect patients’ quality of life [4,5]. The diagnosis of IBD relies on comprehensive evaluation using endoscopic, histological, clinical, and radiological criteria [6]. In practice, colonoscopy plays a vital role in the diagnosis, treatment, and follow-up monitoring of IBD [7,8]. However, since UC and CD have a similar appearance in colonoscopy, endoscopists may misinterpret the images due to limited experience or unconscious bias, resulting in misdiagnosis, missed diagnoses, and delayed treatment [9]. Therefore, it is necessary to improve the IBD classification accuracy to better distinguish between UC and CD in colonoscopy images, which would enhance patients’ quality of life and equip endoscopists with tools that increase both the accuracy and efficiency of clinical care [10].

With the rapid development of artificial intelligence, data-driven deep learning-based approaches have demonstrated impressive performance in computer-aided endoscopic tasks [11,12,13]. However, the lack of labeled data presents a major challenge for deep learning-based IBD classification. Semi-supervised learning, which leverages both labeled and unlabeled data, provides a promising solution to this problem [14,15]. Commonly adopted semi-supervised learning paradigms in endoscopic classification are pseudo-labeling [16] and consistency regularization [17]. In particular, pseudo-labeling-based methods [16] assign pseudo-labels to unlabeled data based on model predictions, which are then used for supervised training. Consistency regularization [17] assumes that different perturbed or augmented versions of the same input should yield consistent predictions. Building on these two paradigms, previous studies combine pseudo-labeling and consistency regularization techniques to achieve impressive performance [18,19,20,21]. However, high intra-class heterogeneity and low inter-class variance among IBD colonoscopy images may lead to inaccurate pseudo-labels. When the model overfits to these inaccurate pseudo-labels, performance degrades due to confirmation bias [22]. Recently, inspired by self-supervised learning approaches [23,24], Yang et al. [25] designed a class-aware contrastive module to learn more separated feature clusters at the class-level to improve model predictions. This method demonstrates promising performance on the semi-supervised classification of natural images. However, under the context of the IBD semi-supervised classification task, it overlooks intra-class heterogeneity. Forcing highly diverse samples within the same class to learn overly aligned representations may suppress fine-grained semantic information, leading to suboptimal classification performance.

To explicitly model intra-class heterogeneity, previous works [26,27] have attempted to decompose each class into multiple subclasses via clustering and then train classifiers with subclass-aware supervision. However, these methods require all samples to have class labels, thus failing to leverage abundant unlabeled data. Additionally, prototype-based learning has been widely adopted in clustering-based self-supervised learning approaches such as SwAV [28] and DeepCluster [29]. These methods learn representations by contrasting predicted cluster assignments of correlated views of the same image. To aggregate samples together while avoiding collapsed solutions, they employ simple clustering-based pseudo-labeling algorithms (e.g., K-means clustering [30] or the Sinkhorn–Knopp algorithm [31]) to generate assignments. Without access to labeled data, these self-supervised methods learn prototypes that do not encode explicit class information, resulting in suboptimal performance when directly applied to classification tasks.

To address these challenges, a **S**ubclass-**A**ware **C**ontrastive **S**emi-**S**upervised **L**earning (SACSSL) framework is proposed, which incorporates a carefully designed subclass-aware contrastive module into a pseudo-labeling-based semi-supervised learning paradigm (i.e., FixMatch [18]). The key innovation of the proposed subclass-aware contrastive module lies in explicitly exploiting abundant unlabeled samples to effectively capture intra-class heterogeneity. Instead of treating each class as a single compact cluster, multiple prototypes per class are introduced to automatically discover fine-grained subclasses in the embedding space. By associating unlabeled samples with different prototypes and enforcing subclass-aware contrastive objectives, the proposed module enhances intra-subclass compactness while preserving rich intra-class diversity, leading to more discriminative and semantically meaningful representations. Specifically, in the subclass-aware contrastive module, unlabeled data are partitioned into confident and uncertain sets based on the maximum probability of their pseudo-labels. Because confident samples with high prediction confidence are more likely to provide reliable supervision, while uncertain samples with low prediction confidence tend to introduce noise and thus require cautious handling, different contrastive objectives are designed to different subsets of unlabeled data to fully exploit their potential. Specifically, an instance-level contrastive loss is applied to encourage the separation of uncertain samples from all other samples in the embedding space to mitigate the confirmation bias in the semi-supervised training. To model the intra-class heterogeneity of IBD images, each class is represented by a set of prototypes in the embedding space. Confident samples are assigned to different prototypes within the same class to form fine-grained subclasses, which allows the supervised contrastive loss [32] to promote intra-subclass clustering among confident samples, effectively enhancing class separability while preserving fine-grained intra-class semantics. The main contributions of the proposed method can be summarized as follows:

- A novel subclass-aware contrastive semi-supervised learning framework for accurate colonoscopy image classification is introduced, which explicitly exploits abundant unlabeled samples to effectively capture intra-class heterogeneity.
- A novel subclass-aware contrastive module that adaptively handles unlabeled data with different contrastive objectives based on pseudo-label confidence is designed, aiming to suppress confirmation bias for uncertain samples and discover fine-grained subclasses for confident samples, thereby enhancing class separability while capturing intra-class variations.
- Comprehensive experiments are conducted on two colonoscopy image datasets, i.e., an in-house collected Daping dataset for IBD classification and a publicly available LIMUC dataset for UC severity grading. The experimental results demonstrate that the proposed method achieves superior classification performance compared to state-of-the-art semi-supervised learning methods on both datasets.

## 2. Related Works

### 2.1. Deep Learning-Based IBD Diagnosis Applications

A variety of deep learning-based approaches have been developed to assist the IBD diagnosis across different modalities. [12,13,33,34,35,36,37,38,39,40,41]. In colonoscopy, Ruan et al. [12] developed a deep learning model based on a deep convolutional neural network to distinguish between UC and CD using over 47,000 endoscopic images from a private dataset, achieving better classification performance compared to junior endoscopists. Similarly, Mauricio et al. [33] developed interpretable deep learning models to classify UC and CD with visualization modules that highlight disease-relevant regions, thereby enhancing clinical trust. In another study, Stidham et al. [34] evaluated the performance of Inception V3, a 159-layer CNN [35], on the task of endoscopic severity grading of UC, showing performance on par with experienced human reviewers. To address the class imbalance commonly observed in IBD-related classification tasks, recent works have proposed specialized techniques, such as high-frequency balancing and augmentation [38] or class distance weighted loss functions [39] to improve minority-class recognition.

Beyond colonoscopy, Klang et al. [36] employed EfficientNet-B5 [37] for detecting strictures from capsule endoscopy images in CD, showing robust generalization capability in multi-center datasets. Similarly, Malik et al. [40] achieved 99.45% accuracy in multi-class classification of UC, polyps, and dyed-lifted polyps from wireless capsule endoscopy (WCE) images using hybrid CNN-GRU architectures. Extending to panenteric capsule endoscopy, Brodersen et al. [41] demonstrated that AI-assisted PCE can effectively differentiate CD, UC, and cancer in a prospective multi-center study, significantly reducing image review time using the AXARO^®^ framework. More recently, Das et al. [42] developed a deep learning model for classifying IBD activity grades from histopathology whole slide images, achieving robust diagnostic performance that could assist pathologists in consistent IBD assessment.

However, these approaches heavily rely on large amount of expert-annotated data that is often difficult to acquire due to the time-consuming annotation process and expertise knowledge requirement.

### 2.2. Semi-Supervised Learning in Endoscopic Image Analysis

Semi-supervised learning has emerged as a promising paradigm by leveraging both labeled and unlabeled data to improve the IBD classification performance, thereby alleviating the heavy reliance on large amount of expert-annotated data. Previous studies have explored the application of semi-supervised learning in endoscopic image analysis [20,21,43,44,45], primarily employing two techniques: pseudo-labeling [16] and consistency regularization [17]. Guo et al. [43] proposed a semi-supervised learning framework with an Adaptive Aggregated Attention module for automatic wireless capsule endoscopy image classification, significantly outperforming supervised baselines. Muruganantham et al. [44] developed ACT-WISE, which employed a teacher–student framework to enforce consistency under perturbations of unlabeled wireless capsule endoscopy images, and integrated active learning for improving label efficiency. There exist other works that combine pseudo-labeling and consistency regularization techniques. Notably, FixMatch [18] integrated both techniques by applying weak and strong augmentations to each unlabeled image, where predictions from the weakly augmented image served as pseudo-labels for training on the strongly augmented image, which has shown impressive classification performance in natural image benchmarks. This has been extended by previous studies [20,21,45] to semi-supervised endoscopic image classification. For example, Huang et al. [20] proposed the class-specific distribution alignment strategy to improve the quality of pseudo-labels when training on highly imbalanced datasets and demonstrate its effectiveness in endoscopic image classification.

Beyond consistency regularization and pseudo-labeling, recent studies have explored incorporating auxiliary tasks into semi-supervised frameworks to enhance representation learning. These tasks can be employed either by self-supervised pretraining to provide better model initialization or incorporating auxiliary objectives into the semi-supervised framework to refine feature representations. For example, Wang et al. [46] proposed a semi-supervised framework that is first pretrained with self-supervised learning on a large unlabeled dataset and then fine-tuned on a limited labeled dataset, achieving promising performance on classifying colorectal neoplasia from narrow-band imaging colonoscopic images under low-label settings. Golhar et al. [47] proposed a novel semi-supervised method that combines an unsupervised jigsaw puzzle solving task with supervised learning, which has achieved promising results in classifying lesions from colonoscopy images. Yang et al. [25] incorporate a class-aware contrastive module into the pseudo-labeling-based semi-supervised framework. Specifically, they apply class-level clustering to in-distribution samples and instance discrimination contrastive loss to out-of-distribution samples, and they achieve impressive performance on natural image benchmarks.

## 3. Materials and Methods

In this work, we consider a semi-supervised IBD classification problem from colonoscopy images. Specifically, for a *C*-class classification problem, let the input image be $x\in R^{H\times W\times 3}$ and the ground truth label be y∈{1,…,C}, where *H* and *W* denote the height and width of the image, and 3 corresponds to the RGB channels. Let $D_{l}=\left\{\left(x_{l}^{n},y_{l}^{n}\right)|n=1,\ldots ,N_{l}\right\}$ and $D_{u}=\left\{x_{u}^{m}|m=1,\ldots ,N_{u}\right\}$ be the labeled and unlabeled datasets, respectively, where $x_{l}^{n}$ and $y_{l}^{n}$ are the labeled images and the corresponding labels, and $x_{u}^{m}$ are the unlabeled images. $N_{l}$ and $N_{u}$ are the number of samples in the labeled dataset and the unlabeled dataset, respectively, where $N_{u}\gg N_{l}$. We employ a visual encoder $g_{\theta }$ to obtain feature representation. We further attach a prediction head $h_{\theta }$ to the visual encoder $g_{\theta }$ to produce distribution over classes, i.e., $p\left(y|x;\theta \right)=h_{\theta }\left(g_{\theta }\left(x\right)\right)$. Moreover, we attach a projection head $h_{\phi }$ to the visual encoder $g_{\theta }$ to obtain low-dimensional features in embedding space for contrastive learning, i.e., $z=h_{\phi }\left(g_{\theta }\left(x\right)\right)$, where *z* represents the projected representation of input image *x*. At each epoch, we first randomly shuffle the labeled dataset $D_{l}$ and the unlabeled dataset $D_{u}$, and then split each dataset into a series of smaller data chunks. We sample one data chunk from the labeled dataset and one from the unlabeled dataset at each iteration. Let B be a data batch which has *B* samples in total, containing labeled data $B_{l}=\left\{\left(x_{l}^{n},y_{l}^{n}\right)|n=1,\ldots ,B_{l}\right\}$ and unlabeled data $B_{u}=\left\{x_{u}^{m}|m=1,\ldots ,B_{u}\right\}$ where $B_{l}$ and $B_{u}$ are the number of labeled samples and the unlabeled samples in the batch, respectively.

**Figure 1:**
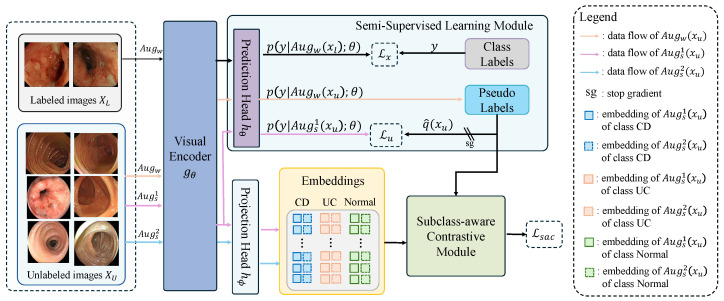
A schematic overview of the proposed SACSSL. The framework comprises a visual encoder, a prediction head, and a projection head. These components are integrated with two complementary modules: a semi-supervised learning module and a subclass-aware contrastive module. See main text for a detailed explanation.

A schematic illustration of the proposed SACSSL is presented in Figure 1. The framework comprises a visual encoder $g_{\theta }$, a prediction head $h_{\theta }$ and a projection head $h_{\phi }$, which are integrated with a semi-supervised learning module and a novel subclass-aware contrastive module. Within the framework, we first apply a weak augmentation $Aug_{w}\left(\cdot \right)$ to all images and two strong augmentations $Aug_{s}^{1}\left(\cdot \right)$ and $Aug_{s}^{2}\left(\cdot \right)$ to the unlabeled images. All augmented images are then passed through the visual encoder to extract features, which are subsequently optimized by two complementary modules. In the semi-supervised learning module, ground-truth labels supervise the weakly augmented labeled samples, while pseudo-labels generated from weakly augmented unlabeled images serve as supervision targets for their first strongly augmented counterparts. Subsequently, embeddings from paired strongly augmented unlabeled images are extracted via the projection head $h_{\phi }$ and jointly processed by the subclass-aware contrastive module. In the subclass-aware contrastive module, confident and uncertain samples are identified based on pseudo-labels, and then tailored contrastive learning strategies are applied to each group to preserve fine-grained intra-class semantics while enhancing inter-class separability, as detailed in Section 3.2. These components are jointly optimized in an end-to-end manner with three objectives: a supervised loss $L_{s}$, an unsupervised loss $L_{u}$, and a subclass-aware contrastive loss $L_{sac}$. Details will be presented below. For a quick reference, we list common symbols used in this section in Table 1.

### 3.1. Semi-Supervised Learning Module

Following FixMatch [18], the semi-supervised learning module consists of two cross-entropy loss terms: a supervised loss $L_{s}$ applied to labeled data and an unsupervised loss $L_{u}$ applied to unlabeled data.

For each labeled sample $\left(x_{l}^{n},y_{l}^{n}\right)$ (where $n\in \{1,\ldots ,B_{l}\})$, we are aiming at minimizing the cross-entropy loss between the model’s predicted distribution over classes on the weakly augmented view $Aug_{w}\left(x_{l}^{n}\right)$ and the corresponding ground truth label $y_{l}^{n}$. Accordingly, the supervised loss $L_{s}$ is defined as

(1) $$L_{s}=\frac{1}{B_{l}}\sum_{n=1}^{B_{l}}H\left(y_{l}^{n},p\left(y|Aug_{w}\left(x_{l}^{n}\right);\theta \right)\right)$$

where H(·,·) denotes the cross-entropy function.

For each unlabeled sample $x_{u}^{m}$ (where $m\in \{1,\ldots ,B_{u}\}$), we generate a weak augmented view $Aug_{w}\left(x_{u}^{m}\right)$ and a strong augmented view $Aug_{s}^{1}\left(x_{u}^{m}\right)$. The soft pseudo-label is derived by computing the model’s predicted distribution over classes on the weakly augmented view, i.e., $q\left(x_{u}^{m}\right)=p\left(y|Aug_{w}\left(x_{u}^{m}\right);\theta \right)$. Subsequently, the hard pseudo-label is obtained by taking the class with the highest predicted probability, i.e., $\hat{q}\left(x_{u}^{m}\right)=\mathrm{arg max}\;q\left(x_{u}^{m}\right)$. To ensure quality of the pseudo-label, we retain only those samples whose highest predicted probability exceeds a threshold *T* for training. Formally, we define a binary indicator: $\eta_{m}=I\left(\max q\left(x_{u}^{m}\right)>T\right)$, where I(·) is the indicator function that returns 1 if the condition holds and 0 otherwise. Finally, we aim to minimize the cross-entropy loss between the hard pseudo-label $\hat{q}\left(x_{u}^{m}\right)$ and the model’s predicted distribution over classes on the strongly augmented view, i.e., $p(y|Aug_{s}^{1}\left(x_{u}^{m}\right);\theta )$. Accordingly, the unsupervised loss $L_{u}$ is defined as

(2) $$L_{u}=\frac{1}{B_{u}}\sum_{m=1}^{B_{u}}\eta_{m}\cdot H\left(\hat{q}\left(x_{u}^{m}\right),p\left(y|Aug_{s}\left(x_{u}^{m}\right);\theta \right)\right)$$

### 3.2. Subclass-Aware Contrastive Module

**Figure 2:**
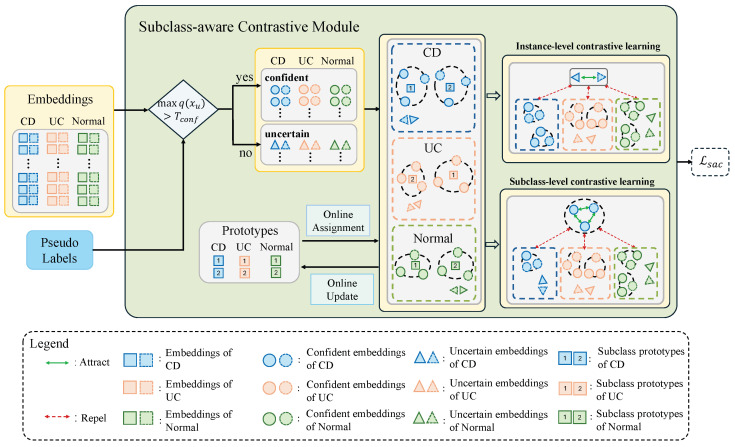
A schematic illustration of the subclass-aware contrastive module. The module processes embeddings from two strong augmentations ($Aug_{s}^{1}$ and $Aug_{s}^{2}$, shown as solid and dashed shapes, respectively) of each image. It first separates embeddings into confident and uncertain groups based on pseudo-label confidence. Confident embeddings are assigned to online-updated subclass prototypes (numbered squares) to form subclasses, where black dashed circles indicate embeddings belonging to the same subclass. Subclass-level contrastive learning then optimizes these confident embeddings by attracting intra-subclass embeddings while repelling inter-subclass ones. Meanwhile, instance-level contrastive learning optimizes uncertain embeddings to repel samples from different images, where gray rectangles enclose augmentation pairs from the same original image. Together, these two components form the subclass-aware contrastive loss.

The limited visual difference between different classes and the significant appearance variability within the same class lead to inaccurate pseudo-labels, which post great challenges for accurate IBD classification. To meet these challenges, we introduce a subclass-aware contrastive module to regularize the low-dimensional feature embedding space. A schematic illustration of the proposed subclass-aware contrastive module is presented in Figure 2. In the subclass-aware contrastive module, confident and uncertain samples are first identified based on the pseudo-labels. To capture intra-class heterogeneity, each class is represented by a set of online-updated prototypes. Confident samples are then assigned to these prototypes to form subclasses, and distinct contrastive objectives are respectively applied to confident and uncertain samples in the embedding space. Specifically, uncertain samples are optimized with an instance-level contrastive loss to reduce the impact of inaccurate pseudo-labels. For confident samples, a subclass-level contrastive loss is designed to encourage subclass-level clustering, enabling the model to preserve fine-grained intra-class semantics and enhance inter-class separability. A step-by-step explanation of the details is presented below.

For each unlabeled sample $x_{u}^{m}\in B_{u}$, we generate a second strongly augmented view $Aug_{s}^{2}\left(x_{u}^{m}\right)$ in addition to the first view $Aug_{s}^{1}\left(x_{u}^{m}\right)$. We then construct two data batches composed of the strong augmented views and the corresponding soft pseudo-labels, which are defined as $B_{u}^{1}=\left\{\left(Aug_{s}^{1}\left(x_{u}^{m}\right),q\left(x_{u}^{m}\right)\right)|m=1,\ldots ,B_{u}\right\}$ and $B_{u}^{2}=\left\{\left(Aug_{s}^{2}\left(x_{u}^{m}\right),q\left(x_{u}^{m}\right)\right)|m=1,\ldots ,B_{u}\right\}$. Subsequently, these batches are merged into a multi-view batch $B_{m}=B_{u}^{1}\cup B_{u}^{2}$, which contains $2B_{u}$ samples. For notational simplicity, we denote the multi-view batch as $B_{m}=\left\{\left({\tilde{x}}_{i},{\tilde{q}}_{i}\right)|i=1,\ldots ,2B_{u}\right\}$, where ${\tilde{x}}_{i}$ represents a strongly augmented view and ${\tilde{q}}_{i}$ is its corresponding soft pseudo-label. Subsequently, all images in the multi-view batch are fed through the visual encoder $g_{\theta }\left(\cdot \right)$ and the projection head $h_{\phi }\left(\cdot \right)$ to produce the embedding set $Z=\{z_{i}|i=1,\ldots ,2B_{u}\}$, where each embedding is $\ell_{2}$-normalized. Next, let $I=\{1,\ldots ,2B_{u}\}$ be the set of indices of all augmented samples within the multi-view batch. We partition *I* into confident and uncertain sets based on the confidence of the corresponding pseudo-label: $I_{conf}=\left\{i\in I|\max\left({\tilde{q}}_{i}\right)>T_{conf}\right\},$ $I_{unc}=\left\{i\in I|\max\left({\tilde{q}}_{i}\right)\leq T_{conf}\right\}$, where $T_{conf}$ is a scalar hyperparameter that determines whether a sample should be considered as a confident sample. This separation allows the model to employ the instance-level contrastive loss $L_{unc}$ and the subclass-level contrastive loss $L_{conf}$ anchored, respectively, at uncertain and confident samples. In the following, we describe the design of the instance-level and the subclass-level contrastive losses in detail.

#### 3.2.1. Instance-Level Contrastive Loss

To mitigate the confirmation bias during training, we employ an instance-level contrastive loss on uncertain samples, following the formulation in SimCLR [23], where each image instance is treated as its own class. Specifically, taking an anchor embedding from the uncertain sample $z_{i}$ (where $i\in I_{unc}$) and another embedding $z_{j}$ (where i≠j) for example, we regard the embedding pair $\left(z_{i},z_{j}\right)$ to be positive only when $z_{j}$ is the embedding from the alternative augmented view of the same original image, which is denoted as $z_{i^{★}}$. Otherwise, the embedding pair is considered to be negative. Subsequently, the instance-level contrastive loss $L_{unc}$ is formulated as

(3) $$\begin{array}{c} L_{unc}=-\sum_{i\in I_{unc}}\log\frac{\exp\left(z_{i}\cdot z_{i^{★}}/\tau \right)}{\sum_{j=1}^{2B_{u}}I_{\left(j\neq i\right)}\exp\left(z_{i}\cdot z_{j}/\tau \right)} \end{array}$$

where τ is a temperature parameter.

#### 3.2.2. Prototype-Based Subclass-Level Contrastive Loss

To capture fine-grained intra-class semantics and improve inter-class separability, we propose a prototype-based subclass-level contrastive learning strategy. Confident samples are assigned to a set of class-specific prototypes, effectively forming subclasses. Inspired by [32], we designed a supervised contrastive loss to encourage samples within each subclass to cluster closely while remaining distinct from other subclasses. The details about the prototype-based subclass-level contrastive loss are presented below.

**Online Prototype Assignment.** For each class c∈{1,…,C}, we incorporate K subclass prototypes ${\left\{p_{k}^{c}\right\}}_{k=1}^{K}$ to represent this class, where each prototype $p_{k}^{c}\in R^{D}$ is an $\ell_{2}$-normalized vector in the embedding space. Let $I^{c}$ denote the indices of confident samples in the multi-view batch that are predicted as class c, i.e., $I^{c}=\left\{\;i|\arg\max{\tilde{q}}_{i}=c,\;i\in I_{conf}\;\right\}$, and let $N_{c}=\left|I^{c}\right|$ denote the number of samples in $I^{c}$. We enumerate these indices as $I^{c}=\left\{i_{1}^{c},\ldots ,i_{N_{c}}^{c}\right\}$. The corresponding confident samples are collected as $X^{c}=\left\{{\tilde{x}}_{i_{n}^{c}}|n\in 1,\ldots ,N_{c}\right\}$. Our objective is to assign the samples in $X^{c}$ to the *K* subclass prototypes of class *c*. We denote the sample-to-prototype mapping as $M^{c}={\left[m_{i_{n}^{c}}\right]}_{n=1}^{N_{c}}\in R^{K\times N_{c}},$ where each $m_{i_{n}^{c}}\in R^{K}$ represents the assignment probabilities of sample $x_{i_{n}^{c}}$ over the *K* prototypes.

Concretely, given the embedding matrix $Z^{c}={\left[z_{i_{n}^{c}}\right]}_{n=1}^{N_{c}}\in R^{D\times N_{c}}$ where each $z_{i_{n}^{c}}\in R^{D}$ is the $\ell_{2}$-normalized embedding of $x_{i_{n}^{c}}$, $M^{c}$ is optimized by solving an optimal transport problem [48] that maximizes the cosine similarity between $Z^{c}$ and the prototypes, i.e., $P^{c}={\left[p_{k}^{c}\right]}_{k=1}^{K}\in R^{D\times K}$ with an entropy regularization term. The optimization objective is defined as

(4) $$\begin{array}{cc} \; & \;\;\max_{M^{c}\in Q}\;\mathrm{Tr}\left(M^{c⊤}P^{c⊤}Z^{c}\right)+\kappa \sum_{i,j}-M_{i,j}^{c}\;\log M_{i,j}^{c},\\ \; & \;s.t.\;M^{c}\in R_{+}^{K\times N_{c}},\;M^{c}1_{N_{c}}=1_{K},\;M^{c⊤}1_{K}=\frac{N_{c}}{K}1_{N_{c}} \end{array}$$

where κ is a parameter controls the smoothness of $M^{c}$, while $1_{K}$ and $1_{N_{c}}$ denote all of the *K*-dimension and $N_{c}$-dimension vectors, respectively. The unique assignment constraint, i.e., $M^{c}1_{N_{c}}=1_{K}$, ensures that each sample is assigned to one prototype. The equipartition constraint, i.e., $M^{c⊤}1_{K}=\frac{N_{c}}{K}1_{N_{c}}$, enforces each prototype is selected at least $\frac{N_{c}}{K}$ times on average in the multi-view batch, which prevents the trivial solution. The solution to this optimization problem is

(5) $$M^{c}=\mathrm{diag}\left(u\right)\;\exp\;\;\left(\frac{{P^{c}}^{⊤}Z^{c}}{\kappa }\right)\;\mathrm{diag}\left(v\right)$$

where $u\in R^{K}$ and $v\in R^{N}$ are renormalization vectors, which can be updated by a few steps of the Sinkhorn–Knopp algorithm [31].

**Prototype-based Subclass-level Contrastive Loss.** With the assignment probability matrix $M^{c}$, we online group the samples $X^{c}$ into *K* prototypes ${\left\{p_{k}^{c}\right\}}_{k=1}^{K}$ within class *c*. After processing all confident samples in the multi-view batch, each confident sample ${\tilde{x}}_{i}$ (where $i\in I_{conf}$) is assigned to $k_{i}$-th prototype of class $c_{i}$, where $c_{i}=\arg\max{\tilde{q}}_{i}$ and $k_{i}=\mathrm{arg max}\;m_{i}$.

Subsequently, taking an anchor embedding from a confident sample $z_{i}$ (where $i\in I_{conf}$) and an another embedding $z_{j}$ (where j≠i) for example, we regard the embedding pair $\left(z_{i},z_{j}\right)$ to be positive if one of the following conditions holds: (1) $z_{j}$ is the embedding from the alternative augmented view of the same original image, which is denoted as $z_{i^{★}}$, or (2) $z_{j}$ corresponds to a different original image but is assigned to the same prototype as $z_{i}$. Formally, we define the set of cross-image positive indices as $Pos\left(i\right)\equiv \left\{\;j\in I_{conf}\;|\;c_{i}=c_{j},k_{i}=k_{j},j\neq i,j\neq i^{★}\;\right\}$. Otherwise, the embedding pair is considered to be negative. To weaken the bias of enforcing alignment between samples with potentially incorrect pseudo-labels, we introduce a confident weight $w_{ip}$ for positive pairs from different images. Specifically, for a positive pair $\left(z_{i},z_{p}\right)$ where p∈Pos(i), the confident weight is defined as $w_{ip}=\max\left({\tilde{q}}_{i}\right)\cdot \max\left({\tilde{q}}_{p}\right)$.

We calculate contrastive loss $L_{conf,i}$ anchored at $z_{i}$ as follows:

(6) $$\begin{array}{cc} L_{conf,i}=\; & -\log\frac{\exp\left(z_{i}\cdot z_{i^{★}}/\tau \right)}{\sum_{j=1}^{2B_{u}}I_{\left(j\neq i\right)}\;\exp\left(z_{i}\cdot z_{j}/\tau \right)}\\ \; & -\sum_{p\in Pos(i)}w_{ip}\cdot \log\frac{\exp\left(z_{i}\cdot z_{p}/\tau \right)}{\sum_{j=1}^{2B_{u}}I_{\left(j\neq i\right)}\;\exp\left(z_{i}\cdot z_{j}/\tau \right)} \end{array}$$

where τ denotes the temperature parameter.

The prototype-based subclass-level contrastive loss $L_{conf}$ is defined by

(7) $$L_{conf}=\sum_{i\in I_{conf}}\frac{1}{1+|Pos(i)|}L_{conf,i}$$

where |Pos(i)| is the number of positive samples for the *i*-th sample in the multi-view batch.

**Prototype Update.** At the training stage, the prototypes ${\left\{p_{k}^{c}\right\}}_{c=1,k=1}^{C,K}$ are updated in an online manner using the embeddings assigned to them. Specifically, after each iteration, each prototype is updated based on the normalized center of its assigned embeddings, which can be formulated as

(8) $$p_{k}^{c}\leftarrow \alpha p_{k}^{c}+\left(1-\alpha \right)\frac{\sum_{z_{i}\in A_{c}^{k}}z_{i}}{\left|\right|\sum_{z_{i}\in A_{c}^{k}}z_{i}{\left|\right|}_{2}}$$

where $A_{c}^{k}$ represents the set of embeddings assigned to prototype $p_{k}^{c}$ in the multi-view batch, and α is the momentum coefficient.

#### 3.2.3. Subclass-Aware Contrastive Loss

The subclass-aware contrastive loss $L_{sac}$ is defined as the average of losses anchored at the confident and the uncertain samples over the multi-view batch.

(9) $$L_{sac}=\frac{1}{2B_{u}}\left(L_{conf}+L_{unc}\right)$$

### 3.3. Overall Objective Function

The overall objective function of the proposed method is

(10) $$L_{total}=L_{s}+\lambda_{1}L_{u}+\lambda_{2}L_{sac}$$

where $\lambda_{1}$ and $\lambda_{2}$ are weighting parameters for the unsupervised loss $L_{u}$ and the subclass-aware contrastive loss $L_{sac}$, respectively.

## 4. Results

### 4.1. Experimental Setting

**Study approval.** This retrospective study was approved by the ethics committee of Daping Hospital affiliated with Army Military Medical University (No. 2018-137). A waiver of informed consent was granted by the same ethics committee. In addition, the clinical study registration number is ChiCTR2100043278. The study complies with the Declaration of Helsinki.

**Datasets.** We conduct comprehensive experiments on two colonoscopy image datasets: an in-house collected Daping dataset for IBD classification and a publicly available LIMUC dataset [49] for UC severity grading.

(1) *Daping dataset:* The Daping dataset comprises 17,161 colonoscopy images from 599 patients collected at the Department of Gastroenterology, Daping Hospital, Army Medical University, between January 2018 and November 2020. Following quality control, images were independently annotated by two experienced gastroenterologists with disagreements resolved by a third expert. Specifically, the Daping dataset consists of three categories, including 1093 CD images, 3379 UC images, and 12,689 normal mucosa images.
(2) *LIMUC dataset:* The LIMUC dataset [49] consists of 11,276 colonoscopy images collected from 564 patients. The images are annotated by experienced gastroenterologists according to the Mayo Endoscopic Score (MES) and are distributed across four severity grades, including 6105 images with MES 0, 3052 images with MES 1, 1254 images with MES 2, and 865 images with MES 3.

We randomly split the dataset by patient into training and testing sets with a ratio of 8:2, ensuring that no images from the same patient appear in both sets. Within the training set, the labeled and unlabeled data are also split by patient to maintain patient-level separation. We adopt a default labeled-to-unlabeled data ratio of 2:8, meaning that 20% of the training samples are labeled while the remaining 80% are treated as unlabeled. The split is also performed at the patient level, ensuring that all images from a single patient are assigned exclusively to either the labeled or the unlabeled subset.

**Implementation Details.** The proposed framework is implemented in PyTorch 2.5.0, and all experiments are performed on one NVIDIA GeForce RTX 4090 GPU and an Intel(R) Xeon(R) Silver 4214R CPU @ 2.40GHz. The network backbone is ViT-B/16-224 [50]. For experiments on the in-house collected Daping dataset, the visual encoder is initialized through self-supervised pretraining on the training set using i-JEPA [51]. For experiments on the publicly available LIMUC dataset [49], we initialize the visual encoder directly with ImageNet-pretrained weights [52] to facilitate reproducibility. It is worth noting that for fair comparison, all competing methods evaluated on the same dataset employ the identical visual encoder with the same initialization strategy. The weak augmentation $Aug_{w}\left(\cdot \right)$ includes random cropping and flipping. We adopt RandAugment [53] as the strong augmentation function $Aug_{s}\left(\cdot \right)$. The training batch sizes for labeled and unlabeled data are 40 and 80, respectively. For the hyperparameters, we empirically set T=0.8, $\lambda_{1}=1$, $\lambda_{2}=2$, $T_{conf}=0.9$, D=128, κ=0.05, K=3, and α=0.99. The AdamW optimizer [54] is employed with a weight decay of $5\times 10^{-4}$. The learning rate is initialized to $10^{-3}$ and linearly decreases to $10^{-6}$ over 150 epochs. The model saved at the end of 150 epochs is used for testing.

**Evaluation Metrics.** We report the classification performance using four evaluation metrics: accuracy, specificity, sensitivity and F1-score. Accuracy is calculated as the proportion of correctly classified samples over the entire test set. In addition, sensitivity, specificity, and F1-score are computed for each class and then averaged across all classes. Specifically, for each class *c*, $TP_{c}$, $TN_{c}$, $FP_{c}$, and $FN_{c}$ denote the true positives, true negatives, false positives, and false negatives, respectively. Then, the evaluation metrics are calculated by

(11) $$\begin{array}{cc} \mathrm{Accuracy} & =\frac{\sum_{c=1}^{C}TP_{c}+TN_{c}}{\sum_{c=1}^{C}TP_{c}+TN_{c}+FP_{c}+FN_{c}} \end{array}$$

(12) $$\begin{array}{cc} \mathrm{Sensitivity} & =\frac{1}{C}\sum_{c=1}^{C}\frac{TP_{c}}{TP_{c}+FN_{c}} \end{array}$$

(13) $$\begin{array}{cc} \mathrm{Specificity} & =\frac{1}{C}\sum_{c=1}^{C}\frac{TN_{c}}{TN_{c}+FP_{c}} \end{array}$$

(14) $$\begin{array}{cc} F1\text{-}\mathrm{score} & =\frac{1}{C}\sum_{c=1}^{C}\frac{2TP_{c}}{2TP_{c}+FP_{c}+FN_{c}} \end{array}$$

### 4.2. Comparison with State-of-the-Art Semi-Supervised Learning Methods

To evaluate performance, we compared our method with five SOTA semi-supervised learning methods on both the Daping dataset and the LIMUC dataset [49]. The characteristics of each SOTA method are summarized as follows. (1) FixMatch [18], which combines pseudo-labeling with consistency regularization, and applies a fixed confidence threshold to ensure the quality of pseudo-labels; (2) FreeMatch [55], which extends FixMatch by adopting an adaptive thresholding strategy that dynamically adjusts the confidence threshold in a class-aware manner, achieving a better quality–quantity trade-off; (3) Class-aware Semi-supervised Contrastive Learning (CCSSL) [25], which integrates a class-aware contrastive module into the FixMatch framework. It separately handles in-distribution data with class-level clustering and out-of-distribution data with instance-level contrastive; (4) SoftMatch [56], which extends FixMatch by using a truncated Gaussian weighting function to assign confidence-based weights to unlabeled samples rather than using a fixed threshold to filter them; (5) Semantic-aware FixMatch (SA-FixMatch) [21], which replaces the standard random CutOut in FixMatch’s strong augmentation with a semantic-aware CutOut.

Moreover, to quantify the performance of different competing methods, we trained two additional models on the dataset. These two models had the same network architecture as our method. The first model, referred to as the upper bound, was trained on 100% labeled data in a fully supervised manner, while the second model, referred to as the baseline, was trained in a fully supervised manner on 20% labeled data without using any unlabeled data.

To assess the statistical significance of performance differences between models, we apply bootstrap resampling [57] to estimate the distribution of pairwise differences in F1-score, as the F1-score provides a more reliable overall metric than accuracy for imbalanced classification. A 95% confidence interval (CI) of the difference is then computed. Following established statistical interpretation [58,59], when a confidence interval (CI) includes zero, it means that the observed difference is not statistically significant at the 95% confidence level.

**Results on the Daping Dataset.** The quantitative comparison with the competing SOTA methods when using 20% labeled data for training is presented in Table 2. From this table, one can observe that our method achieves the best classification performance with an accuracy of 93.2%, a sensitivity of 76.8% and an F1-score of 80.1%. Specifically, our method outperforms the second-best method (CCSSL [25]) by a margin of 0.5%, 0.8% and 1.9% in terms of accuracy, sensitivity and F1-score, respectively. Moreover, the 95% CI of the F1-score difference is [0.003, 0.041], which does not include zero, indicating that the performance improvement of our method over CCSSL [25] is statistically significant at the 95% confidence level for the semi-supervised IBD classification task. The experimental results demonstrate the effectiveness of the proposed method in the semi-supervised IBD classification of colonoscopy images. Additionally, from Table 2, one can observe that our proposed SACSSL surpasses the baseline performance by a substantial margin of 2.2%, 2.6%, 1.0% and 4.6% in terms of accuracy, sensitivity, specificity and F1-score, respectively, and it is close to the upper-bound performance, with a small gap of 0.8%, 1.6%, 1.5% and 0.7% in terms of accuracy, sensitivity, specificity and F1-score, respectively. These results demonstrate the superior capability of our method in leveraging the unlabeled data for the semi-supervised IBD classification of colonoscopy images.

**Results on the LIMUC Dataset.** The quantitative comparison with the competing SOTA methods when using 20% labeled data for training is presented in Table 3. From this table, one can observe that our method achieves the highest accuracy (76.4%) and F1-score (68.9%) along with a sensitivity of 67.7% and a specificity of 91.0%. In comparison, CCSSL [25] attains a higher sensitivity of 68.9% and a specificity of 91.2% but with lower accuracy (75.9%) and F1-score (68.0%). The 95% CI of the F1-score difference between our method and CCSSL [25] is [−0.005, 0.026], indicating that the performance improvement is not statistically significant at the 95% confidence level. This outcome may be attributed to the inherent characteristics of the UC severity grading task, which consists of fine-grained categories with smaller inter-class differences and more constrained intra-class variation, naturally limiting the potential gains achievable by the subclass-aware contrastive module. Despite these constraints, our method still achieves the highest overall accuracy and F1-score. The experimental results demonstrate the effectiveness of the proposed method in semi-supervised UC severity grading, indicating its adaptability across different IBD-related colonoscopy image classification tasks.

### 4.3. Analytical Ablation Studies

We further conduct analytical ablation studies on the Daping dataset to investigate the effectiveness of different components of the proposed method. In particular, we conduct the following analytical ablation studies: (1) We first evaluate the classification performance of the proposed method using different percentages of labeled data; (2) We then investigate the effectiveness of each loss to the overall classification performance gain in the proposed method; (3) We further investigate the influence of the confidence threshold $T_{conf}$ and the number of prototypes per class *K* in the subclass-aware contrastive module on the performance of the proposed method; (4) Additionally, we investigate the influence of the visual encoder backbone on the performance of the proposed method; (5) Finally, we conduct an analysis of learned features.

**Evaluation under Different Percentages of Labeled Data.** We conducted an ablation study to investigate the impact of the percentage of labeled data on the performance of the proposed method. We compared our method with the baseline model when 5%, 10%, 20% and 30% labeled data were used for training. In particular, the baseline model was trained in a supervised manner using only the labeled data, while our method was trained in a semi-supervised manner using both labeled and unlabeled data. The experimental results are presented in Table 4. As shown in this table, our method demonstrates consistent classification performance improvement under all proportions of labeled data compared to the baseline performance. Notably, when trained with only 5% labeled data, our method outperforms the baseline performance by a large margin of 4.7%, 5.6%, and 6.0% in terms of accuracy, specificity and F1-score, respectively, demonstrating its effectiveness in leveraging unlabeled data under very limited labeled data. As the proportion of labeled data increases to 30%, our method outperforms the baseline performance by a margin of 1.9%, 4.4%, 1.4%, and 4.9% in terms of accuracy, sensitivity, specificity, and F1-score, respectively. These results demonstrate the superior capability of our method in leveraging the unlabeled data to improve classification performance.

**Effectiveness of Different Losses.** To validate the effectiveness of different losses used in our method, we conduct an ablation study by training the model with different combinations of losses using 20% labeled data: (1) $L_{s}$; (2) $L_{s}+L_{u}$; (3) $L_{s}+L_{u}+L_{unc}$; (4) $L_{s}+L_{u}+L_{conf}$; and (5) $L_{s}+L_{u}+L_{sac}$ (where $L_{sac}$ is a combination of $L_{unc}$ and $L_{conf}$). The experimental results are reported in Table 5. Compared to the baseline model trained with $L_{s}$ alone, the model trained with $L_{s}$ and $L_{u}$ improves the classification performance by a margin of 1.0% in terms of accuracy, while showing a performance drop by a margin of 3.1% and 1.1% in terms of sensitivity and F1-score, respectively, which could be potentially attributed to confirmation bias. In addition to $L_{u}$ and $L_{s}$, incorporating $L_{unc}$ further improves the accuracy, sensitivity, specificity and F1-score by a margin of 0.4%, 4.2%, 0.8% and 2.9%, respectively, which demonstrates the effectiveness of the instance-level contrastive loss when applied to uncertain samples. Similarly, incorporating $L_{conf}$ in addition to $L_{u}$ and $L_{s}$ improves the classification performance by a margin of 0.5%, 2.0%, 0.5% and 1.5% in terms of accuracy, sensitivity, specificity and F1-score, respectively, demonstrating the effectiveness of the subclass-level contrastive loss when applied to confident samples. Finally, the model that incorporates all the losses results in the best classification performance, outperforming the second-best classification performance by a margin of 0.7%, 1.5%, 0.2%, and 2.8% in terms of accuracy, sensitivity, specificity, and F1-score, respectively, which demonstrates the two complementary components of our subclass-aware contrastive loss $L_{sac}$, composed of $L_{unc}$ and $L_{conf}$, provide synergistic benefits for representation learning. These results demonstrate that the subclass-aware contrastive loss, derived from our proposed subclass-aware contrastive module, effectively enhances representation learning and improves the overall classification performance.

**Influence of the Confidence Threshold $T_{\mathrm{conf}}$.** The confidence threshold $T_{conf}$ determines the separation of confident and uncertain samples in our method. A low $T_{conf}$ may include samples with incorrect pseudo-labels in the confident set, introducing noise to subclass-level contrastive learning, whereas a high $T_{conf}$ may exclude correctly pseudo-labeled samples from the confident set, which weakens the effectiveness of subclass-level contrastive learning. To investigate the influence of the confidence threshold, we conduct an ablation study by setting $T_{conf}$ to a value in {0.6,0.7,0.8,0.9,0.95}. The experimental results are reported in Table 6. From this table, one can observe that the model achieves the best classification performance when $T_{conf}$ is set to 0.9 with an accuracy of 93.2% and an F1-score of 80.1%. Therefore, we adopt $T_{conf}=0.9$ in our study.

**Influence of the Number of Prototypes per Class K.** The number of prototypes per class *K* controls the subclass granularity within each multi-view batch. Too few prototypes may fail to capture fine-grained intra-class variations in IBD colonoscopy images, while too many prototypes may push semantically similar samples away in the embedding space, reducing the effectiveness of contrastive learning. Here, we conduct an ablation study to investigate the performance of the proposed method when setting different *K* values, where *K* is in {2,3,4,5,6}. The experimental results are reported in Table 7. From this table, the model achieves the best classification performance when *K* is set to 3 with an accuracy of 93.2% and an F1-score of 80.1%. Therefore, we adopt K=3 in our study.

**Influence of the Visual Encoder Backbone.** The choice of visual encoder backbone directly affects the quality of extracted visual features, which in turn influences classification performance. To evaluate this effect, we conduct an ablation study using three widely adopted models as the visual encoder backbone: two CNN-based models, ResNet50 [60] and EfficientNet-B5 [37], and a transformer-based model, ViT-B [50]. The experimental results are summarized in Table 8. From this table, one can observe that when using ViT-B [50] as the visual encoder, our method achieves the best classification performance, outperforming the best CNN-based backbone (ResNet-50 [60]) by a margin of 1.7%, 0.1%, 1.1%, and 2.5% in terms of accuracy, sensitivity, specificity, and F1-score, respectively. These results indicate that the transformer-based backbone consistently outperforms the CNN-based backbones in the semi-supervised colonoscopy image classification task. Accordingly, we adopt ViT-B as the visual encoder backbone in our study.

**Figure 3:**
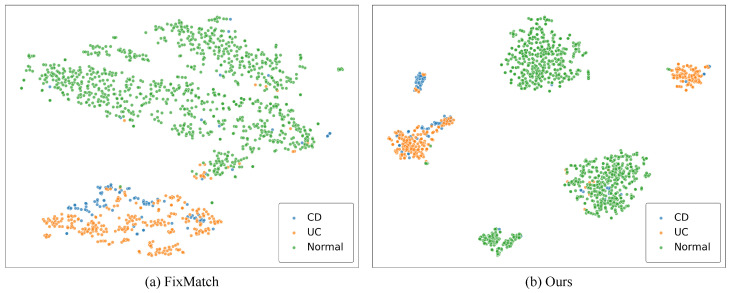
Comparison of the t-SNE visualization of the embedded feature distributions on the Daping test set between FixMatch [18] and the proposed SACSSL using t-SNE. Each color denotes one of the 3 classes.

**Analysis of the Learned Features.** To validate the effectiveness of our method in representation learning, we use the t-SNE algorithm [61] to visualize the distributions of learned features extracted from the visual encoder $g_{\theta }$ by projecting the embedded features into a two-dimensional space. In Figure 3, we present a qualitative comparison of the t-SNE visualization on the Daping test set between FixMatch [18] and the proposed SACSSL. The t-SNE analysis is implemented in Python using the scikit-learn package [62] (version 1.5.1). Each point in Figure 3 represents the embedded feature from one colonoscopy image and is color-coded using the ground truth class label. From this figure, one can see that the classification boundary learned by FixMatch, especially between UC and CD, is unclear, such that it is difficult to correctly classify IBD from colonoscopy images. By incorporating our proposed subclass-aware contrastive module, SACSSL learns feature representations with clearer inter-class boundaries, while intra-class features are further partitioned into multiple compact clusters according to their semantic information. This explains why the proposed SACSSL achieves superior classification performance.

## 5. Discussion

The accurate classification of IBD from colonoscopy images is critical for timely diagnosis and effective treatment. Although deep learning-based approaches have achieved impressive performance in various medical image classification tasks, developing robust models for IBD diagnosis remains challenging due to the limited availability of annotated endoscopic data and the severe intra-class heterogeneity of colonoscopy images. Semi-supervised learning methods provide a promising alternative to this problem by leveraging both labeled and unlabeled data. In particular, the pseudo-labeling-based framework combined with consistency regularization [18,55,56] has proven to be an effective and simple approach. CCSSL [25] extended pseudo-labeling-based frameworks by refining learned features through class-aware contrastive learning that promotes intra-class clustering. Although this approach performs well on natural image tasks, it struggles to capture the substantial intra-class heterogeneity of colonoscopy images in IBD classification. However, prior approaches for modeling intra-class heterogeneity either rely on fully supervised subclass decomposition that cannot exploit unlabeled data or adopt class-agnostic prototype learning in a self-supervised manner, making it difficult to simultaneously leverage unlabeled data and capture fine-grained intra-class variations for IBD classification. To address these limitations, we propose SACSSL, which incorporates the proposed subclass-aware contrastive module into the FixMatch framework. This design enables us to refine learned features by applying an instance-level contrastive loss to distinguish uncertain samples and applying a subclass-level contrastive loss to confident samples to learn hierarchical clustered representations within each class. We evaluate our method on an in-house collected colonoscopy image dataset for IBD classification, which is referred to as the Daping dataset. The proposed method outperforms the SOTA methods, as presented in Table 2. As demonstrated in Table 4, our method achieves consistently improved performance compared to the baseline performance regardless of the percentage of labeled data, which demonstrates that our model can effectively leverage unlabeled data. Moreover, we further evaluate our method on the publicly available LIMUC dataset for UC severity grading, which is an IBD-related classification task. As shown in Table 3, our method outperforms other competing SOTA methods, demonstrating its effectiveness on UC severity grading as an additional colonoscopy image classification task.

The key to the superior performance of SACSSL lies in the carefully designed subclass-aware contrastive module. In particular, we leverage subclass prototypes to partition confident samples into subclasses and apply supervised contrastive loss to learn hierarchical, clustered representations within each class. Quantitative and qualitative results from our analytical ablation studies conducted on the Daping dataset demonstrate that SACSSL learns better-separated feature representations with the proposed subclass-aware contrastive module, as demonstrated by Figure 3 and Table 5.

The clinical implications of the proposed method merit further discussion. In clinical practice, large amounts of colonoscopy data are readily available, whereas obtaining expert annotations remains costly and time consuming. By effectively leveraging abundant unlabeled data available in clinical archives, the proposed framework alleviates the heavy reliance on expert annotations, which is one of the major barriers to the real-world deployment of deep learning models. Moreover, the improved inter-class separability and preserved intra-class diversity achieved through subclass-aware contrastive learning enhance the model’s capability to distinguish between UC and CD, which is a clinically important yet challenging task even for experienced endoscopists. Collectively, these advantages highlight the potential clinical impact of the proposed method in supporting large-scale, annotation-efficient colonoscopy image diagnosis.

In addition, several limitations of the current study, as well as potential directions for future research, deserve further discussion. First, although the proposed method achieves consistently superior performance on both the in-house Daping dataset and the publicly available LIMUC dataset, the current evaluation is still limited to single-center data. However, differences in patient populations, imaging devices, and acquisition protocols across institutions may introduce domain shifts, which could potentially lead to degraded performance on external datasets. Recent studies [63,64,65] have shown that semi-supervised domain adaptation techniques can effectively mitigate such discrepancies by leveraging labeled source data and unlabeled target domain data, which provides a promising direction for extending the proposed framework to multi-center scenarios. Second, the present work follows a closed-set semi-supervised learning setting, where all categories in the unlabeled data are assumed to be known. However, in real-world clinical environments, unlabeled data may inevitably contain unknown categories. Recent advances in open-set and universal semi-supervised learning [21,66] offer potential solutions by explicitly handling unknown samples during training. In future studies, integrating such strategies into the proposed framework could further enhance its robustness in practical scenarios. Third, the hyperparameters in this study were selected empirically. Although the current configuration already yields superior performance compared to other competing SOTA methods, more systematic hyperparameter optimization strategies, such as grid search, could be explored in future work to potentially achieve further performance gains.

## 6. Conclusions

In this paper, we propose a novel subclass-aware contrastive semi-supervised learning framework for accurate colonoscopy image classification, which is referred to as SACSSL. The proposed framework features a novel subclass-aware contrastive module, which explicitly captures intra-class heterogeneity by adaptively applying instance-level contrastive learning to uncertain samples and prototype-based subclass-level contrastive learning to confident samples, thereby enhancing inter-class separability while preserving fine-grained intra-class diversity and mitigating confirmation bias. Comprehensive experiments on both the in-house Daping dataset and the public LIMUC dataset demonstrate that SACSSL consistently outperforms existing semi-supervised learning methods in both IBD classification and UC severity grading. In addition, the t-SNE analysis further confirms that SACSSL is capable of learning clear classification boundaries while preserving fine-grained intra-class semantics, highlighting its effectiveness in IBD classification from colonoscopy images. In future work, we will focus on extending the proposed framework to semi-supervised domain adaptation, aiming to improve robustness across centers with different endoscopy systems and patient populations. Moreover, we will explore the universal semi-supervised learning method to explicitly handle non-IBD or atypical colonoscopic findings that commonly appear in large-scale unlabeled clinical data.

## Figures and Tables

**Table 1:** Common symbols used in Section 3.

Symbol	Meaning	Sec.
$x_{l}^{n},y_{l}^{n}$	Labeled image and the corresponding ground truth label	Section 3
$x_{u}^{m}$	Unlabeled image	Section 3
$g_{\theta }$	Visual encoder	Section 3
$h_{\theta }$	Prediction head	Section 3
$h_{\phi }$	Projection head	Section 3
p(y|x;θ)	Model’s predicted distribution over classes	Section 3
*z*	Projected representation in embedding space	Section 3
$B_{l},B_{u}$	Labeled and unlabeled subsets in a data batch	Section 3
$B_{l},B_{u}$	Number of labeled and unlabeled samples in a data batch	Section 3
$Aug_{w}\left(\cdot \right)$	Weak augmentation	Section 3
$Aug_{s}^{1}\left(\cdot \right)$	First strong augmentation	Section 3
$Aug_{s}^{2}\left(\cdot \right)$	Second strong augmentation	Section 3
q(·)	Soft pseudo-label	Section 3.1
$\hat{q}\left(\cdot \right)$	Hard pseudo-label	Section 3.1
$B_{m}$	Multi-view batch	Section 3.2
${\tilde{x}}_{i},{\tilde{q}}_{i}$	Strongly augmented view and its soft pseudo-label	Section 3.2
$T_{conf}$	Confidence threshold for separating confident and uncertain samples	Section 3.2
$I_{conf}$	Index set of confident samples	Section 3.2
$I_{unc}$	Index set of uncertain samples	Section 3.2
$I^{c}$	Index set of confident samples predicted as class *c*	Section 3.2.2
$p_{k}^{c}$	*k*-th prototype of class *c*	Section 3.2.2
$M^{c}$	Sample-to-prototype assignment probability matrix for class *c*	Section 3.2.2
$Z^{c}$	Embedding matrix for confident samples of class *c*	Section 3.2.2
$P^{c}$	Prototype matrix containing *K* prototypes for class *c*	Section 3.2.2

**Table 2:** Quantitative comparison with SOTA semi-supervised learning methods on the Daping dataset. The best results are highlighted in bold. LDP: percentage of labeled data; UDP: percentage of unlabeled data.

Method	Percentage (%)		Metrics (%)			
	LDP	UDP	Accuracy	Sensitivity	Specificity	F1-Score
Upper-Bound	100	0	94.0	78.4	96.4	80.8
Baseline	20	0	91.0	74.2	93.9	75.5
FixMatch [18]	20	80	92.0	71.7	93.9	74.4
FreeMatch [55]	20	80	92.2	73.8	94.2	76.1
CCSSL [25]	20	80	92.7	76.0	**95.0**	78.2
SoftMatch [56]	20	80	92.3	74.9	93.9	77.1
SA-FixMatch [21]	20	80	91.7	74.5	94.4	76.5
**Ours**	20	80	**93.2**	**76.8**	94.9	**80.1**

**Table 3:** Quantitative comparison with SOTA semi-supervised learning methods on the LIMUC dataset [49]. The best results are highlighted in bold. LDP: percentage of labeled data; UDP: percentage of unlabeled data.

Method	Percentage (%)		Metrics (%)			
	LDP	UDP	Accuracy	Sensitivity	Specificity	F1-Score
Upper-Bound	100	0	79.6	74.4	92.2	73.4
Baseline	20	0	72.1	62.3	89.2	63.2
FixMatch [18]	20	80	75.1	65.9	90.4	66.9
FreeMatch [55]	20	80	75.2	68.2	90.8	68.7
CCSSL [25]	20	80	75.9	**68.9**	**91.2**	68.0
SoftMatch [56]	20	80	76.1	67.4	91.1	67.3
SA-Fixmatch [21]	20	80	75.7	66.8	90.3	67.8
**Ours**	20	80	**76.4**	67.7	91.0	**68.9**

**Table 4:** Performance of our method on the Daping dataset under different percentages of labeled data (%). The best results are highlighted in bold font. LDP: labeled data percentage; UDP: unlabeled data percentage.

Method	Percentage (%)		Metrics (%)			
	LDP	UDP	Accuracy	Sensitivity	Specificity	F1-Score
Upper-Bound	100	0	94.0	78.4	96.4	80.8
Baseline	5	0	85.6	70.8	86.8	65.3
Ours	5	95	**90.3**	**68.5**	**92.4**	**71.3**
Baseline	10	0	89.2	73.5	92.2	71.1
Ours	10	90	**92.2**	**75.0**	**94.6**	**77.1**
Baseline	20	0	91.0	74.2	93.9	75.5
Ours	20	80	**93.2**	**76.8**	**94.9**	**80.1**
Baseline	30	0	91.5	73.0	94.1	74.9
Ours	30	70	**93.4**	**77.4**	**95.5**	**79.8**

**Table 5:** Results on validating the effectiveness of different losses. The best results are highlighted in bold font. LDP: labeled data percentage; UDP: unlabeled data percentage.

Method	Loss Functions				Percentage (%)		Metrics (%)			
	$L_{s}$	$L_{u}$	$L_{\mathrm{unc}}$	$L_{\mathrm{conf}}$	LDP	UDP	Accuracy	Sensitivity	Specificity	F1-Score
Upper-Bound	✓				100	0	94.0	78.4	96.4	80.8
Baseline	✓				20	0	91.0	74.2	93.9	75.5
$L_{s}+L_{u}$	✓	✓			20	80	92.0	71.1	93.9	74.4
$L_{s}+L_{u}+L_{unc}$	✓	✓	✓		20	80	92.4	75.3	94.7	77.3
$L_{s}+L_{u}+L_{conf}$	✓	✓		✓	20	80	92.5	73.1	94.4	75.9
Ours	✓	✓	✓	✓	20	80	**93.2**	**76.8**	**94.9**	**80.1**

**Table 6:** Results on investigating the influence of the confidence threshold $T_{conf}$. The best results are highlighted in bold. LDP: percentage of labeled data; UDP: percentage of unlabeled data.

$T_{\mathrm{conf}}$	Percentage (%)		Metrics (%)			
	LDP	UDP	Accuracy	Sensitivity	Specificity	F1-Score
0.6	20	80	92.1	**78.5**	94.8	78.8
0.7	20	80	93.0	77.9	**95.3**	79.7
0.8	20	80	92.9	76.4	95.1	78.7
0.9	20	80	**93.2**	76.8	94.9	**80.1**
0.95	20	80	92.3	74.6	94.0	77.5

**Table 7:** Results on investigating the influence of the number of prototypes per class *K*. The best results are highlighted in bold. LDP: percentage of labeled data; UDP: percentage of unlabeled data.

*K*	Percentage (%)		Metrics (%)			
	LDP	UDP	Accuracy	Sensitivity	Specificity	F1-Score
2	20	80	92.7	73.3	94.7	76.2
3	20	80	**93.2**	**76.8**	94.9	**80.1**
4	20	80	92.7	74.6	94.9	77.3
5	20	80	92.5	73.7	94.7	76.4
6	20	80	92.9	74.2	**95.0**	77.2

**Table 8:** Results on investigating the influence of architecture of the visual encoder $f_{\theta }$. The best results are highlighted in bold. LDP: percentage of labeled data; UDP: percentage of unlabeled data.

Backbone	Percentage (%)		Metrics (%)			
	LDP	UDP	Accuracy	Sensitivity	Specificity	F1-Score
ResNet-50 [60]	20	80	91.5	76.6	93.8	77.6
EfficientNet-B5 [37]	20	80	92.1	72.9	94.1	75.5
ViT-B [50]	20	80	**93.2**	**76.8**	**94.9**	**80.1**

## Data Availability

The original contributions presented in this study are included in the article. Further inquiries can be directed to the corresponding author.

## Abbreviations

The following abbreviations are used in this manuscript:

IBD	Inflammatory bowel disease
CD	Crohn’s disease
UC	Ulcerative colitis
WCE	Wireless capsule endoscopy
PCE	Panenteric capsule endoscopy
MES	Mayo Endoscopic Score
CI	Confidence interval
